# Coping with kidney disease – qualitative findings from the Empowering Patients on Choices for Renal Replacement Therapy (EPOCH-RRT) study

**DOI:** 10.1186/s12882-017-0542-5

**Published:** 2017-04-03

**Authors:** Lalita Subramanian, Martha Quinn, Junhui Zhao, Laurie Lachance, Jarcy Zee, Francesca Tentori

**Affiliations:** 1 Arbor Research Collaborative for Health, 340 E. Huron, Suite 300, Ann Arbor, MI 48104 USA; 2 The Center for Managing Chronic Disease, University of Michigan, 1415 Washington Heights, Ann Arbor, MI 48109 USA; 3 Vanderbilt University Medical Center, 1211 Medical Center Drive, Nashville, TN 37232 USA

**Keywords:** Coping strategies, CKD, Peritoneal dialysis, Hemodialysis, CSI

## Abstract

**Background:**

The highly burdensome effects of kidney failure and its management impose many life-altering changes on patients. Better understanding of successful coping strategies will inform patients and help health care providers support patients’ needs as they navigate these changes together.

**Methods:**

A qualitative, cross-sectional study involving semi-structured telephone interviews including open- and closed-ended questions, with 179 U.S. patients with advanced chronic kidney disease (CKD), either not yet on dialysis ([CKD-ND], *n* = 65), or on dialysis (hemodialysis [HD], *n* = 76; or peritoneal dialysis [PD], *n* = 38) recruited through social media and in-person contacts from June to December 2013. Themes identified through content analysis of interview transcripts were classified based on the Coping Strategies Index (CSI) and compared across groups by demographics, treatment modality, and health status.

**Results:**

Overall, more engagement than disengagement strategies were observed. “Take care of myself and follow doctors’ orders,” “accept it,” and “rely on family and friends” were the common coping themes. Participants often used multiple coping strategies. Various factors such as treatment modality, time since diagnosis, presence of other chronic comorbidities, and self-perceived limitations contributed to types of coping strategies used by CKD patients.

**Conclusions:**

The simultaneous use of coping strategies that span different categories within each of the CSI subscales by CKD patients reflects the complex and reactive response to the variable demands of the disease and its treatment options on their lives. Learning from the lived experience of others could empower patients to more frequently use positive coping strategies depending on their personal context as well as the stage of the disease and associated stressors. Moreover, this understanding can improve the support provided by health care systems and providers to patients to better deal with the many challenges they face in living with kidney disease.

## Background

At the end of 2013, there were 655,435 people in the United States with kidney failure, an increase of 68% since 2000 [1]. Treatment options for kidney failure include kidney transplantation (2.6% of incident patients in 2013) and dialysis (97.2%) [1]. Chronic kidney disease (CKD) requires a multi-faceted treatment plan that impacts many aspects of everyday life, including diet, fluid intake, time available for leisure activities, and self-management of multiple therapeutic interventions and medications [2]. CKD patients often also have to deal with several other chronic comorbidities, especially diabetes, hypertension, and cardiovascular diseases [3, 4]. These patients also face the challenge and burden of the intrusive and irreversible consequences of kidney failure on their physical and mental health, as well as on their quality of life, including the impact on their family, lifestyle, relationships, and employment [5, 6].

The clinical effects of kidney failure include fluid retention, anemia, elevated blood pressure, bone and mineral disorders, accelerated cardiovascular disease, and sexual dysfunction [7, 8]. The effects of kidney failure and its treatment, the lack of a cure, and the impact of treatment options on lifestyle and well-being contribute to a sustained source of stressors in the lives of these patients [2].

While CKD onset is most common later in life (mean age 62.5 years in the United States [1]), some patients develop kidney failure in childhood, resulting in a lifetime of managing the disease and its impact on their lives.

Coping is a response mechanism used to regulate the effect of different types of life stressors on physiological responses [9]. There is some evidence that coping strategies employed by individuals in dealing with chronic diseases could help explain some differences in their disease survival rates, as well as their ability to adjust to the challenges experienced while living with chronic diseases [10]. For example, a recent study suggests that optimism may have a direct physiological effect on the neuroendocrine system and on immune responses, while also having an indirect effect on health outcomes by promoting protective health behaviors, adaptive coping strategies, and enhancing positive mood [11]. A qualitative study of Thai CKD patients in California suggests that spirituality or religiosity is an important means of coping for these patients [12].

**Fig. 1:**
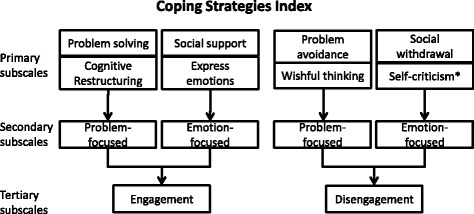
Schematic of the classification of primary, secondary and tertiary subscales within the Coping Strategies Index. *This primary subscale was not identified in our data

Tobin et al. proposed that there is a hierarchical structure to coping strategies (Fig. 1) [13]. These are described as the primary, secondary, and tertiary subscales in the Coping Strategies Inventory (CSI) that has since been used to develop patient-reported outcome measures for coping [9, 14]. At the primary level, this factor structure includes eight coping strategies [13]. At the secondary level, each of the primary categories is classified into either problem-focused coping or emotion-focused coping. Problem-focused coping relates to actions that are taken to alter the source of stress, while emotion-focused coping addresses the emotional distress or the emotional response associated with the source of stress [15]. Coping efforts are categorized at the tertiary level as either engagement or disengagement strategies. Engagement involves confronting stressors and is believed to reduce the impact of long-term physiological and psychological stress. Disengagement strategies rely on avoiding undesirable stressors which often have short-term benefits, but might cause longer-term problems. The CSI questionnaires developed based on this framework are widely used to assess coping in various contexts [9, 16–20]. A prior analysis of CSI data from an international cohort of HD patients found that in the US engagement strategies were used more often than disengagement strategies. However, PD and CKD patients may face different sets of challenges. Close-ended survey responses are valuable but do not provide the richness of information, such as how these coping strategies are being applied in the context of the challenges faced by these patients. Additionally, survey questions could potentially prompt a response with no opportunity to qualify it.

The Empowering Patients on Choices for Renal Replacement Therapy (EPOCH-RRT) Study, supported in part by the Patient-Centered Outcomes Research Institute (PCORI), was designed to empower kidney disease patients by providing relevant information that was systematically derived from other patients’ experiences to promote shared decision making on dialysis modalities. The primary aim of the EPOCH-RRT study was to develop a dialysis decision aid, that is now publicly available (www.choosingdialysis.org). The current work leverages data collected during EPOCH-RRT and describes the results of the analysis through the lens of the CSI framework to investigate how patients cope with kidney disease so as to inform health care professionals and to support patients in managing their chronic conditions.

## Methods

### Study design

The EPOCH-RRT study was developed to understand the decision making process in selecting treatment options from the perspective of the CKD patient. It involved a collaborative approach between academic researchers and an advisory panel (AP) of nine patients and family members, as well as health care professionals (nephrologists, social workers). Kidney disease patients participating on the AP co-designed and pilot-tested the interview protocols [21], met periodically throughout the study to provide input on the analysis, and helped to interpret findings as it related to the development of the decision aid. Protocols used a mixed-methods approach comprising primarily open-ended and some closed-ended questions, including yes/no, categorical, and Likert-type scales (1–10). Participant information on demographics, clinical history, and patients’ perception of their health status were also collected at the time of the interview. Interviewers were trained and followed the interview protocol, in both content and sequence, to ensure uniform data collection.

### Recruitment of participants

Inclusion criteria were: (a) age > 18; (b) eGFR <25 mL/min/1.73 m^2^ or on dialysis (HD or PD) for at least three months. Individuals who had previously had a kidney transplant were not excluded. A total of 181 interviews were conducted and 180 were included in this analysis; one interview was excluded because the participant was the only one receiving care outside of the U.S., where clinical practices, social, and cultural factors may be different. Participants were recruited both through nationwide web and social media (Facebook) outreach and in-person by study team social workers in Southeast Michigan renal clinics and dialysis units, as described previously [21]. At the time of the interviews, 65 participants were CKD-ND, 77 were on HD (65 in-center and 12 home), and 38 were on PD. All study procedures were approved by local institutional review boards (Ethical and Independent Review Services E&I #13016, Henry Ford Health Systems IRB #8144, University of Michigan IRBMED HUM00073058), as appropriate.

### Data collection

The semi-structured interview protocol included questions and probes on experience living with kidney disease, aspects they found most bothersome, how they dealt or coped with their problems, and their decision making involvement with treatment options [21]. Between June and December 2013, two trained interviewers conducted telephone interviews that were digitally recorded and transcribed. Each interview lasted 30-45 min.

### Data analysis methods

Of the 180 interviews, 179 people had responded to the question, “How do you cope or deal with your kidney problems?” All responses were manually coded by two independent coders to identify common themes using content analysis. Coders discussed and resolved discrepancies to achieve consensus. During the analysis, a codebook of theme categories was created, shared among coders, and added to as new themes emerged directly from patient responses. Coders conferred multiple times regarding patterns and themes until no new themes were identified and saturation was reached. Each theme was counted only once for each individual even if it was identified in more than one context. Some themes were identified based on the tone and attitude underlying the response rather than the specific quote, e.g., in a few (2-3) cases where the respondent joked about their condition, the coders agreed, based on the larger context of the response, that these patients were using humor to cope with their disease. Responses were entered into NVivo 10 and common themes were identified across all patients. Once consensus was reached, the final list of 38 themes was classified based on the CSI framework into primary subscales, from which the secondary and tertiary scales were derived. Similarly, themes were generated from all the responses from the same group of patients to the question, “Now I’d like you to think about your kidney problems in general, what bothers you most about having kidney disease?”

## Results

### Demographics

CKD-ND patients tended to be older (mean ages in CKD-ND = 63.4, HD = 55.8, PD = 50.4), had more female respondents, were more frequently diagnosed within the last year, more often felt they were in poor health, more frequently expressed limitations in daily life, and more often had three or more chronic conditions compared to HD and PD patients. More CKD-ND and HD patients were recruited in the study than PD patients (Table 1). Some had prior dialysis experience different from current treatment status.

**Table 1:** Characteristics of participants by treatment modality and attributes at the time of interviews

Attributes	CKD-ND	HD	PD	All
Number of patients, N	65	76	38	179
Age, years, %				
< 45	15.4	32.9	34.2	26.8
45-59	15.4	31.6	34.2	26.3
60-74	36.9	19.7	28.9	27.9
75	32.3	15.8	2.6	19.0
Gender, %				
Male	33.8	52.6	47.4	44.7
Female	66.2	47.4	52.6	55.3
Race/Ethnicity^a^, %				
Caucasian/White	53.8	50.7	60.0	53.7
African American/Black	40.0	44.0	31.4	40.0
Other	6.2	5.3	8.6	6.3
When did you first find out you had kidney problems, %				
Within the past yr	16.9	5.3	0.0	8.4
1-5 years ago	30.8	29.3	31.6	30.3
6-10 year ago	18.5	12.0	21.1	16.3
10+ yrs ago	26.2	37.3	34.2	32.6
Since birth or childhood	7.7	16.0	13.2	12.4
Self-rated health status, %				
Excellent	4.6	5.3	5.3	5.0
Very good	12.3	18.4	21.1	16.8
Good	36.9	40.8	50.0	41.3
Fair	33.8	31.6	21.1	30.2
Poor	12.3	3.9	2.6	6.7
Limitations in daily activities, %				
Yes	49.2	72.0	84.2	66.3
No	50.8	28.0	15.8	33.7
Number of Other Chronic Conditions, %				
0	9.2	6.6	2.6	6.7
1	16.9	15.8	34.2	20.1
2	21.5	32.9	21.1	26.3
3 or more	52.3	44.7	42.1	46.9

^a^1 HD and 3 PD patients did not provide race/ethnicity information

#### Bothersome aspects of a kidney disease diagnosis

The most commonly eluded to bothersome aspect of having kidney disease was the time spent on dialysis. Dialysis was perceived as a limitation by those already on dialysis and dreaded by the CKD-ND patients. One HD patient said, *“The time spent there [dialysis center]. I’m not excited about those sixteen hours; I could use ‘em doing something else.”* Patients were also bothered by diet restrictions, inability to travel freely, the lifelong, incurable, and fatal aspect of the diagnosis as well as the inability to control disease progression. For example, one participant said*, “I think renal patients probably have one of the strictest diets that exist… most of the stuff I like, you know, I’m not supposed to eat.”* Meanwhile, for another patient, what bothered them most was*, “So it’s just the- the fact that this thing won’t go away, it’s my whole life, it’s always there.”* Several patients alluded to the wait for a kidney transplant, as one patient declared what was most bothersome was, *“waiting for so long, on the list that I’m on, for a kidney.”* Patients were troubled by aspects of the disease that impacted their daily life like employment and independence; some were bothered about not having taken care of themselves prior to the diagnosis, loss of control, limited treatment options, symptoms, financial concerns, physical appearance, and social acceptance. One participant described it as, *“something that bothers me is that my family doesn’t understand and I can’t explain it to them in words because there are none to describe each day’s random changes to the condition.”* Responses to this question clearly indicate that kidney disease has a serious impact on the life of patients and these patients have to deal with many long-term physical, psychosocial, and financial stresses.

#### Coping themes and classification of themes

**Fig. 2:**
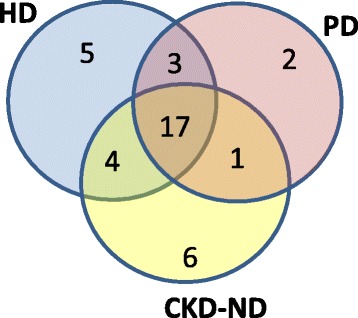
Number of coping themes identified by patients, classified by treatment modality – hemodialysis (HD), peritoneal dialysis (PD) and pre-dialysis (CKD-ND)

The distribution of coping themes across HD, PD, and CKD-ND patients is described in Fig. 2. Seventeen of the themes were common to all three sub-groups; however, each sub-group also had some distinct coping themes. For example, the theme “Compartmentalize and only deal with what I have to at that time” and “Perspective - realize it could it be worse” were two themes that were only identified among PD patients. A PD patient dealing with three or more chronic conditions said, *“I think I compartmentalize. I deal with what has to be dealt with, and then push it aside, when I’ve dealt with it. So it doesn’t interfere with my day-to-day life.”*

“Helping others with kidney disease,” “staying preoccupied during dialysis,” and “be aware of your own limits” were common themes that were identified exclusively among HD patients. There were six themes identified only among CKD-ND patients, and each was unique to a particular patient. All the themes were then classified based on the CSI framework. None of the themes fit under self-criticism, and therefore only seven primary subscales were used. The most frequently identified themes for each of the primary subscales and examples of quotes for these themes are described in Table 2. “Take care of myself and follow doctors’ orders,” “accept it,” and “rely on family and friends” were the most frequently used coping strategies overall. Individuals sometimes used multiple coping themes that were either in the same primary subscale or across different primary subscales. For example, a female CKD-ND patient when asked how she coped said, *“I take a Celexa every day and I try not to take Xanax. I have point- oh-point-two-five, I think it is Xanax and she said try to keep it to not more than two a day. And I try to spread it out over the week that I only take about three times… And I have god and I have a fantastic family. If it wasn’t for that I don’t know what I would do.”*

**Table 2:** Classification of emergent themes from data to the CSI framework

Sample quotes	Representative themes	Primary	Secondary	Tertiary
“I like to crack jokes… make people laugh …when I go there for 4 h I got a whole new audience to impress!”	Keep my sense of humor or use humor	Express emotion	EFE	ENGAGEMENT
“If I didn’t have my wife, I probly [sic] wouldn’t be alive… I’m coping…because of her.” “I have a lot of family around me so that helps. You know, they’re always there for me.”	Rely on family or friends for support and encouragement	Social support		
“If I didn’t have God, I know I wouldn’t be able to deal with it.” “Well, I’ve gotten more spiritual now… attending church more.”	Spiritual or pray			
“I have to accept it, for what it is. I just, accept it and try to live a productive life.”	Accept it	Cognitive restructuring	PFE	
“I can still try to do the best that I can right now.”	Do the best I can			
“You’ve got to keep your access sites clean and …I’ve worked pretty hard at that… And I try not to get colds and …I’ve tried to stay healthy”	Take care of myself and follow doctor’s orders (e.g. medications, diet, fluid, rest)	Problem solving		
“I like hobbies….[listed several hobbies], I learned to crochet” “I still want to build a company, I still want to make money…I could retire …but it’s the last thing on my mind.”	Stay active and busy with hobbies or work			
“I have to be very careful, like I don’t use my cane … I don’t…let them see how vulnerable I am.”	Protect myself and not let others see how vulnerable I am	Social withdrawal	EFD	DISENGAGEMENT
“I don’t think of it in terms of kidney problems, I suspect I probably should”	Ignore it	Problem avoidance	PFD	
“It’s not a problem…I don’t see where I have to cope, I just go through life.”	Feel fine, don’t need a way to cope			
“I hope…to get better…the doctor says that it wouldn’t get any better so I guess the dialysis is …the way to keep you alive.”	Resigned but hope it will get better	Wishful thinking		

#### Primary subscales by demographics

Table 3 describes the percentage of individuals in different demographic groups relying on coping strategies (themes) classified based on the CSI framework. A decrease in percentage of individuals using social support to cope with their kidney disease was observed with increasing age. Within this primary subscale, those less than 45 years most frequently used “rely on family and friends” and “spiritual/pray” themes. For example, a young male on PD said, *“my faith has helped me to accept things.”* More women used social support (34%), with the biggest difference being in the “talk with others about my feelings” theme, and cognitive restructuring (55%) than men (28% and 45%, respectively). Meanwhile, a slightly higher percentage of men (49% vs. 42%) resorted to problem avoidance, typically with the “ignore it” theme, than women. A greater percentage of black patients cited cognitive restructuring strategies (57%), especially “keep going,” than any of the other primary subscales. Among those resorting to disengagement strategies, white patients most often referenced the “ignore it” theme and more frequently used problem avoidance. Other races cited disengagement strategies distributed across several themes including “try not to focus on it,” “I don’t worry about it,” “don’t have a way of dealing with it,” and “feel fine, don’t need a way to cope.” Social support was a more commonly used strategy among non-whites (>36%) than white patients (24%); in particular, the “spiritual/pray” theme was referenced by 24% of black participants compared to 4% by white participants.

**Table 3:** Relative use of coping strategies (themes) classified within primary subscales and by age, gender, and race/ethnicity

Tertiary	Secondary	Primary	Age, %				Gender, %		Race/ethnicity, %		
			<45	45-59	60-74	75+	Male	Female	White	Black	Other
			N = 48	N = 47	N = 50	N = 34	N = 80	N = 99	N = 94	N = 70	N = 11
ENGAGEMENT	EFE	Express emotion	6	2	6	12	6	6	7	4	9
		Social support	46	36	26	12	28	34	24	40	36
	PFE	Cognitive restructuring	50^a^	51^a^	56^a^	41	45	55^a^	44	57^a^	64^a^
		Problem solving	19	15	16	32	20	19	22	16	45
DISENGAGEMENT	EFD	Social withdrawal	0	0	0	3	0	1	0	1	0
	PFD	Problem avoidance	35	53	40	56^a^	49^a^	42	56^a^	33	27
		Wishful thinking	0	2	0	3	1	1	0	1	0

Note: Four people are missing race/ethnicity information..^a^-Most frequently used primary subscale within each subgroup

#### Network of coping strategies

**Fig. 3:**
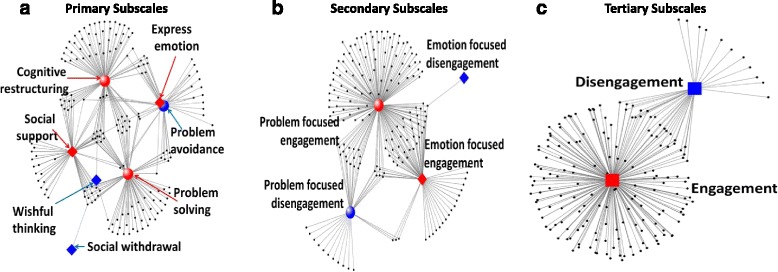
Network of coping strategies used by individuals, by subscales. Black dots represent individuals. Lines connect individuals (black dots) to one or more of the engagement (red square, diamonds, and red circles) and disengagement (blue square, diamonds, and blue circles) strategies used by that person within each of the coping subscales (Panels: **a** – Primary; **b**-Secondary; **c**- Tertiary). In panels **a** and **b**, circular nodes represent problem-focused strategies (red circles: problem solving, cognitive restructuring; blue circles: problem avoidance, wishful thinking) and diamonds represent emotion-focused strategies (red diamonds: social support, express emotion; blue diamond: social withdrawal). In panel **c**, the red square represents all engagement strategies while blue represents all disengagement strategies

Many individuals used multiple coping strategies, spanning both problem-focused and emotion-focused strategies and sometimes within the same primary subscale or across different primary subscales. The linkages across primary subscales were complex, as illustrated in Fig. 3, panel a. Some individuals used strategies that could be classified under three distinct primary subscales.

Problem solving and cognitive restructuring strategies, both problem-focused engagement strategies, predominated and included considerable overlap with social support. This is further highlighted in Panel B, with the majority of individuals linked to the problem-focused engagement subscale with some overlap with other secondary subscales. Only seven individuals used strategies that crossed more than two secondary subscales. Engagement strategies were far more frequently used for coping compared to disengagement strategies, with less than 10% using disengagement strategies exclusively (Fig. 3, Panel c).

#### Coping strategies by current treatment modality

We compared coping strategies by the three treatment modality sub-groups, HD, PD, and CKD-ND (Table 4). HD patients relied more heavily on social support compared to CKD-ND and PD patients. HD patients used fewer types of disengagement strategies, with no individuals using strategies that were classified as either social withdrawal or wishful thinking. CKD-ND patients, at the individual level, used the most diverse set of coping strategies, with at least one or more individuals using each of the primary subscale categories.

**Table 4:** Comparison of coping strategies classified within the primary subscale used by percentage of patients based on treatment modality at the time of interviews

	Modality			All
	CKD-ND	HD	PD	
Express emotion, %	6.2	9.2	0.0	6.1
Social support, %	21.5	40.8	28.9	31.3
Cognitive restructuring, %	50.8	46.1	57.9	50.3
Problem solving, %	53.8	42.1	36.8	45.3
Social withdrawal, %	1.5	0.0	0.0	0.6
Problem avoidance, %	20.0	18.4	21.1	19.6
Wishful thinking, %	1.5	0.0	2.6	1.1
N	65	76	38	179

#### Coping strategies by time since kidney disease diagnosis, number of chronic conditions, and self-reported health limitations

Some interesting differences were observed at the secondary subscale level based on time since kidney disease diagnosis. Overall, strategies classified as problem-focused engagement (PFE), comprising cognitive restructuring and problem solving, were most common across all groups (73%–85%). For example, a 75 year old female patient was classified as using problem-focused engagement based on her response, *“I go religiously every month for my lab work and we have a wonderful doctor and nurse. The next morning you can call in and get a read-out on the lab, renal lab work.”* Use of emotion-focused engagement strategies was more variable across different sub-groups, with those diagnosed with kidney disease between 1 and 10 years ago tending to rely less (<30% of patients) on emotion-focused engagement strategies (EFE) compared to 38%–48% in the other groups. Additionally, those who had been diagnosed with kidney disease 6 years ago or earlier were less likely to use disengagement coping strategies. To further investigate factors that might separate the use of engagement versus disengagement strategies, coping strategies were compared across people with zero, one, two, three, and four or more other chronic conditions. The data suggest that those with no other chronic condition were more likely to rely only on engagement strategies (100%) to cope with their disease and very few resorted to any disengagement strategies (8.3%). A larger proportion, typically around 20%, of patients with one or more chronic conditions, used disengagement strategies.

To explore whether coping strategies vary based on the perceived impact of patients’ health condition on daily life, we compared coping strategies by responses to limitation on daily activities. Both those who answered “yes” as well as those who answered “no” used problem solving and cognitive restructuring strategies. However, those who felt that there were limitations on their daily activity tended to rely more on social support (37.3%) than those who did not (20.0%). Meanwhile, those who felt no limitations in daily activity were more likely to use the disengagement strategy of problem avoidance (26.7%) than those who did feel that their daily activities had been limited by their kidney disease (16.1%). This observation is consistent with those who reported their health status to be “poor,” among whom there was greater use of social support and less frequent use of problem avoidance strategies compared with all other groups.

## Discussion

There are no established standards for addressing personal stressors and supporting effective coping strategies for patients dealing with kidney failure and its long-term impacts on their lives and lifestyles. Other studies suggest that the diet and fluid restrictions, psychosocial stressors such as loss of independence and social stigma, pill burden and depression, fatigue, financial burden, travel limitations, and impact on employment and relationships force CKD patients, both prior to and after starting dialysis, to develop individual strategies to navigate the changes that the disease demands of them [4, 6, 12, 22–25]. Patients with kidney failure often feel better after starting dialysis and this is reflected in our patient cohort where CKD-ND patients more often felt they were in poor health than those on dialysis. The interviews with patients in the EPOCH-RRT study provided additional evidence that there are various sources of bother and stress associated with the disease and treatment choices. The aspects of the disease that these patients found most bothersome align well with other published work, including the prevalence of a perception that external factors, “powerful others,” control favorable outcomes [26], which is reflected in themes such as, “loss of control,” “incurable, fatal, lifelong,” “no control over disease progression,” and “lack of progress in technology and treatment” in this study. A higher score on internal locus of control, i.e., perceived personal control over favorable life and health outcomes, was shown to be positively associated with mental quality of life components among hemodialysis patients using the respective survey instruments [27].

The use of the CSI framework in this study allowed structured categorization of a broad spectrum of coping strategies that then facilitated comparison among sub-groups as well as with results from other studies. Our results suggest that individuals adapt to their circumstances by independently developing coping strategies and often use multiple strategies, spanning different CSI primary subscales, to deal with the complexities of their condition. An interesting finding was that among all the patients interviewed, no theme that could be categorized as self-criticism emerged. It could be that even if patients are self-critical, at times, they may not consider this as a strategy that they have developed to cope with the challenges they face. This subtle, yet important distinction, may not have been apparent if participants had been surveyed using the CSI questionnaire. The most frequently cited themes of “take care of myself and follow doctors’ orders” and “rely on family and friends” suggest that these patients want to take better care of themselves and needed to rely on others to cope with this chronic condition. This may indicate that these patients would be responsive to opportunities for improving self-reliance if they had more structured and systematic support from health care providers and social services to address their quality of life and lifestyle needs, resulting in better patient-centered health outcomes.

Specific types of coping strategies have been proposed as having beneficial effects on health outcomes [15, 28]. In looking across different diseases, the correlation of specific CSI primary subscales to physical and psychological outcomes is variable [29]. For example, some studies have suggested a negative correlation between emotion-focused coping or avoidance strategies, and the mental component of the Quality of Life questionnaire [30, 31] or adherence to fluid restriction [32]. Some reports suggest that HD patients predominantly use denial and avoidance strategies but had similar health outcomes, anxiety, and mood profiles to other dialysis patients [33]. There are also variations in how coping strategies are categorized. For example, we categorized “having” social support as emotion-focused coping while others have considered “seeking” social support as a problem-focused coping strategy among male hemodialysis patients [34]. Additionally, in our exploration of coping themes, there was considerable use of emotion-focused engagement strategies, often combined with problem-focused strategies, by the same person. Therefore, by using a qualitative approach, a deeper understanding has been obtained on the context of these coping strategies among patients with kidney disease and could lead to evaluating the effectiveness of specific coping strategies in dealing with stressors and on outcomes of interest in this patient population [6, 35]. Our work offers insight into the complex network of coping strategies employed by patients with kidney disease and how they relate to the coping strategies index, the theoretical construct from which many of the self-reported quantitative instruments assessing coping strategies are derived. By using a qualitative inductive process to classify the breadth of strategies used by patients, we were able to identify and highlight the diversity of coping strategies within each CSI subscale, with rich information on what each coping strategy looked like from the perspective of these patients as well as at different stages of disease progression. Patients who have lived with a diagnosis of kidney disease for more than a year might cope differently from those recently diagnosed. Furthermore, different coping strategies might be more effective based on where they are in the mental adjustment to chronic illness. [36, 37]. Our results suggest different coping strategies were used by patients not on dialysis, those on hemodialysis, and those on peritoneal dialysis. Time since diagnosis of kidney disease, presence of other comorbidities, and self-perceived limitations from the diagnosis were associated with the types of coping strategies used by patients, especially the proportion of patients using disengagement strategies. Other studies among kidney disease patients have reported a correlation between disengagement strategies and worse clinical outcomes, including mortality [38], emphasizing the importance of understanding coping skills among this patient community. By collecting qualitative data from a large cohort of participants representing a heterogeneous mixture of attributes, the complex network of coping strategies, relevant to their kidney disease context, used by these patients has been highlighted. The patients who participated in this study predominantly used engagement strategies to cope, suggesting greater involvement in the management of their condition and associated life changes. There are some indications that those dealing with multiple comorbidities might more often resort to disengagement strategies to cope with their condition.

A potential limitation to our study is that participants tended to be younger, had higher education levels, and included a higher percentage of females and African Americans compared to the U.S. national population for each modality. The selected study sample is in part the result of our recruitment method using social media, and in-person recruitment being concentrated in a specific geographic region. Given their willingness and ability to participate in lengthy telephone interviews, participants were potentially healthier and more engaged compared to the general U.S. population of CKD and dialysis patients. These factors might be reflected in the high use of engagement rather than disengagement strategies among our participants. However, these same factors may have allowed participants to better articulate their experiences and provide us with better insight on positive coping strategies. Wisdom from those who have successfully coped with their disease could be helpful for others in similar circumstances. Further, the variety of coping techniques and complexity of their interactions observed through this study highlights the many opportunities for the health care system to support individuals in developing effective coping skills and better manage the considerable impact of kidney failure on patient life and well-being. This understanding will help care providers to tailor the coping support to individual’s current needs. For example, being aware that there is a risk for pre-dialysis patients to cope using disengagement strategies such as wishful thinking, health care providers could be more vigilant to this risk and proactively suggest engagement strategies that other patients have described here, such as learning more about the disease and staying active. This might involve facilitating access to or customizing education in problem-solving strategies that could benefit patients dealing with negative symptoms that they might be experiencing; referrals to therapists who could help these patients find appropriate tools for coping might also be an option. Furthermore, connecting patients to the experiences of similarly situated patients could lead to a mutually supportive and engaged community of patients empowered with positive coping skills to deal with the many challenges of living with chronic kidney disease.

## Conclusions

Kidney failure has a serious impact on the lives of patients and these patients have to deal with many long-term physical, psychosocial, and financial stresses. Patients described, in their own words, ways in which they deal with these stressors. Analysis of these responses highlight the complex and reactive coping strategies that patients adopt to the variable demands at different stages of contending with the disease and its treatment options. This qualitative approach illuminates how the CSI framework applies to the context of coping with CKD. Such description of the lived experience of others coping with kidney disease could empower patients with coping skills that suit their individual needs with the knowledge that they have been effective for others in similar situations. Meanwhile, this understanding could improve targeted support provided by health care systems and providers so patients can better deal with the many challenges they face in living with kidney disease.

## Abbreviations

AP: Advisory panel
CKD: Chronic kidney disease
CKD-ND: Not yet on dialysis
CSI: Coping strategies index
EFE: Emotion-focused engagement strategies
EPOCH-RRT: Empowering Patients on Choices for Renal Replacement Therapy
HD: Hemodialysis
PCORI: Patient-centered outcomes research institute
PD: Peritoneal dialysis
PFE: Problem-focused engagement

## References

[1] United States Renal Data System (2016). 2016 USRDS annual data report: Epidemiology of kidney disease in the United States.

[2] Clarke AL, Yates T, Smith AC, Chilcot J (2016). Patient's perceptions of chronic kidney disease and their association with psychosocial and clinical outcomes: a narrative review. Clin Kidney J.

[3] Ronksley PE, Hemmelgarn BR (2012). Optimizing care for patients with CKD. Am J Kidney Dis.

[4] Kahn LS, Vest BM, Madurai N, Singh R, York TR, Cipparone CW, Reilly S, Malik KS, Fox CH (2015). Chronic kidney disease (CKD) treatment burden among low-income primary care patients. Chronic Illn.

[5] Tong A, Sainsbury P, Chadban S, Walker RG, Harris DC, Carter SM, Hall B, Hawley C, Craig JC (2009). Patients’ experiences and perspectives of living with CKD. Am J Kidney Dis.

[6] Reid C, Seymour J, Jones C (2016). A thematic synthesis of the experiences of adults living with hemodialysis. Clin J Am Soc Nephrol.

[7] Ahmad MM, Al Nazly EK (2015). Hemodialysis: stressors and coping strategies. Psychol Health Med.

[8] Mitch W, Goldman L, Ausiello D (2007). Chronic kidney disease. Text book of medicine.

[9] Addison CC, Campbell-Jenkins BW, Sarpong DF, Kibler J, Singh M, Dubbert P, Wilson G, Payne T, Taylor H (2007). Psychometric evaluation of a coping strategies inventory short-form (CSI-SF) in the Jackson heart study cohort. Int J Environ Res Public Health.

[10] Reynolds P, Hurley S, Torres M, Jackson J, Boyd P, Chen VW (2000). Use of coping strategies and breast cancer survival: results from the black/white cancer survival study. Am J Epidemiol.

[11] Avvenuti G, Baiardini I, Giardini A (2016). Optimism’s explicative role for chronic diseases. Front Psychol.

[12] Chatrung C, Sorajjakool S, Amnatsatsue K (2015). Wellness and religious coping among thai individuals living with chronic kidney disease in Southern California. J Relig Health.

[13] Tobin DL, Holroyd KA, Reynolds RV, Wigal JK (1989). The hierarchical factor structure of the coping strategies inventory. Cogn Ther Res.

[14] Addison CC, Campbell-Jenkins BW, Sarpong DF, Kibler J, Singh M, Dubbert P, Wilson G, Payne T, Taylor H (2007). Psychometric evaluation of a coping strategies inventory short-form (CSI-SF) in the Jackson heart study cohort. Int J Environ Res Public Health.

[15] Carver CS, Scheier MF, Weintraub JK (1989). Assessing coping strategies: a theoretically based approach. J Pers Soc Psychol.

[16] Blount RL, Simons LE, Devine KA, Jaaniste T, Cohen LL, Chambers CT, Hayutin LG (2008). Evidence-based assessment of coping and stress in pediatric psychology. J Pediatr Psychol.

[17] Fernandez CA, Loucks EB, Arheart KL, Hickson DA, Kohn R, Buka SL, Gjelsvik A (2015). Evaluating the effects of coping style on allostatic load, by sex: the Jackson Heart Study, 2000–2004. Prev Chronic Dis.

[18] Delfino JP, Barragán E, Botella C, Braun S, Bridler R, Camussi E, Chafrat V, Lott P, Mohr C, Moragrega I, Papagno C, Sanchez S, Seifritz E, Soler C, Stassen HH (2015). Quantifying insufficient coping behavior under chronic stress: a cross-cultural study of 1,303 students from Italy, Spain and Argentina. Psychopathology.

[19] Mackner LM, Crandall WV (2005). Oral medication adherence in pediatric inflammatory bowel disease. Inflamm Bowel Dis.

[20] Speyer E, Morgenstern H, Hayashino Y, Kerr PG, Rayner H, Robinson BM, Pisoni RL (2016). Reliability and validity of the coping strategy inventory-short form applied to hemodialysis patients in 13 countries: results from the dialysis outcomes and practice patterns study (DOPPS). J Psychosom Res.

[21] Dahlerus C, Quinn M, Messersmith E, Lachance L, Subramanian L, Perry E, Cole J, Zhao J, Lee C, McCall M, Paulson L, Tentori F (2016). Patient perspectives on the choice of dialysis modality: results from the empowering patients on choices for renal replacement therapy (EPOCH-RRT) study. Am J Kidney Dis.

[22] Palmer SC, Hanson CS, Craig JC, Strippoli GF, Ruospo M, Campbell K, Johnson DW, Tong A (2015). Dietary and fluid restrictions in CKD: a thematic synthesis of patient views from qualitative studies. Am J Kidney Dis.

[23] Shahgholian N, Yousefi H (2015). Supporting hemodialysis patients: a phenomenological study. Iran J Nurs Midwifery Res.

[24] Ghimire S, Castelino RL, Lioufas NM, Peterson GM, Zaidi ST (2015). Nonadherence to medication therapy in haemodialysis patients: a systematic review. PLoS ONE.

[25] Yu J, Ng HJ, Nandakumar M, Griva K (2016). The management of food cravings and thirst in hemodialysis patients: a qualitative study. J Health Psychol.

[26] Kohli S, Batra P, Aggarwal HK (2011). Anxiety, locus of control, and coping strategies among end-stage renal disease patients undergoing maintenance hemodialysis. Indian J Nephrol.

[27] Birmelé B, Le Gall A, Sautenet B, Aguerre C, Camus V (2012). Clinical, sociodemographic, and psychological correlates of health-related quality of life in chronic hemodialysis patients. Psychosomatics.

[28] Folkman S, Moskowitz JT (2000). Positive affect and the other side of coping. Am Psychol.

[29] Penley JA, Tomaka J, Wiebe JS (2002). The association of coping to physical and psychological health outcomes: A meta-analytic review. J Behav Med.

[30] Pucheu S, Consoli SM, D'Auzac C, Français P, Issad B (2004). Do health causal attributions and coping strategies act as moderators of quality of life in peritoneal dialysis patients?. J Psychosom Res.

[31] Takaki J, Nishi T, Shimoyama H, Inada T, Matsuyama N, Kumano H, Kuboki T (2005). Possible interactive effects of demographic factors and stress coping mechanisms on depression and anxiety in maintenance hemodialysis patients. J Psychosom Res.

[32] O'Connor SM, Jardine AG, Millar K (2008). The prediction of self-care behaviors in end-stage renal disease patients using Leventhal's Self-Regulatory Model. J Psychosom Res.

[33] Nowak Z, Wańkowicz Z, Laudanski K (2015). Denial defense mechanism in dialyzed patients. Med Sci Monit.

[34] Cormier-Daigle M, Stewart M (1997). Support and coping of male hemodialysis-dependent patients. Int J Nurs Stud.

[35] Tong A, Craig JC (2016). Tuning into qualitative research-a channel for the patient voice. Clin J Am Soc Nephrol.

[36] Gilbar O, Or-Han K, Plivazky N (2005). Mental adjustment, coping strategies, and psychological distress among end-stage renal disease patients. J Psychosom Res.

[37] Watson M, Greer S, Young J, Inayat Q, Burgess C, Robertson B (1988). Development of a questionnaire measure of adjustment to cancer: the MAC scale. Psychol Med.

[38] Wolf EJ, Mori DL (2009). Avoidant coping as a predictor of mortality in veterans with end-stage renal disease. Health Psychol.

